# Elevated Bile Acid Is Associated with Worsened Impaired Glucose Homeostasis in Pancreatic Ductal Adenocarcinoma Patients with Extrahepatic Cholestasis through Increased Hepatic Insulin Clearance

**DOI:** 10.3390/jcm12062352

**Published:** 2023-03-17

**Authors:** Jie Yang, Chunlu Tan, Zhenjiang Zheng, Xing Wang, Xubao Liu, Yonghua Chen

**Affiliations:** Division of Pancreatic Surgery, Department of General Surgery, West China Hospital of Sichuan University, Chengdu 610041, China

**Keywords:** pancreaticoduodenectomy, pancreatic ductal adenocarcinoma, extrahepatic cholestasis, impaired glucose homeostasis, bile acid, hepatic insulin clearance

## Abstract

Background: Patients after pancreaticoduodenectomy (PD) showed improved glucose tolerance. Evidence for the effect of extrahepatic cholestasis on impaired glucose homeostasis secondary to ductal adenocarcinoma of the pancreatic head is limited. Methods: In this prospective cross-sectional study, 50 patients with ductal adenocarcinoma of the pancreatic head were included to assess the effect of extrahepatic cholestasis on glucose tolerance status based on the oral glucose tolerance test (OGTT) before pancreatic surgery. Results: Patients with extrahepatic cholestasis more frequently suffered from worsened impaired glucose homeostasis (prediabetes and new-onset diabetes, 95.2% vs. 58.6%, *p* = 0.004). Elevated bile acid level was recognized as an independent risk factor for impaired glucose homeostasis (*p* = 0.024, OR = 6.85). Hepatic insulin clearance (HIC) was significantly higher in patients with elevated bile acid levels (*p* = 0.001). A strong positive correlation was found between bile acid levels and HIC (r = 0.45, *p* = 0.001). Conclusions: This study suggested a connection between elevated bile acid levels and worsened impaired glucose homeostasis through increased insulin clearance function in ductal adenocarcinoma of pancreatic head patients.

## 1. Introduction

Pancreatic ductal adenocarcinoma (PDAC) is one of the most identified causes of type 3c diabetes mellitus, which is defined as diabetes secondary to diseases of the exocrine pancreas [1]. In contrast to type 1 and type 2 diabetes, diabetes secondary to PDAC incorporates causes of diabetes with different pathophysiology. Previous works have shown that the local effects of tumor infiltration, pancreatic ductal obstruction, and probably a paraneoplastic effect due to mediators released by cancer cells could consequently cause beta-cell dysfunction and insulin resistance [2].

Recent clinical studies have found that patients after pancreaticoduodenectomy (PD) showed improved glucose tolerance; however, this phenomenon is not observed in patients after distal pancreatectomy [3,4,5,6,7,8]. Different from distal pancreatic tumors, extrahepatic cholestasis is one of the manifestations of ductal adenocarcinoma of the pancreatic head [9]. Indeed, in cholangiocarcinoma patients and pregnancies with non-diabetic histories, the development of extrahepatic or intrahepatic cholestasis is associated with impaired glucose tolerance [10,11,12]. Moreover, bile acids, bilirubin, and lipids, which are metabolically altered after extrahepatic cholestasis, could affect glucose metabolism [13,14,15]. Thus, we assumed that one of the reasons for the release from impaired glucose tolerance after PD might be the resolution of extrahepatic cholestasis. For impaired glucose homeostasis secondary to ductal adenocarcinoma of the pancreatic head before pancreatic surgery, evidence for the effect of extrahepatic cholestasis on glucose metabolism is limited.

In this prospective cross-sectional study, patients with ductal adenocarcinoma of the pancreatic head were included to assess the effect of extrahepatic cholestasis on glucose tolerance statuses and the relationship between impaired glucose homeostasis and the alteration of bile acid metabolism. Furthermore, the alterations of glycemic traits were evaluated by another OGTT after PD.

## 2. Materials and Methods

### 2.1. Study Design

**Figure 1:**
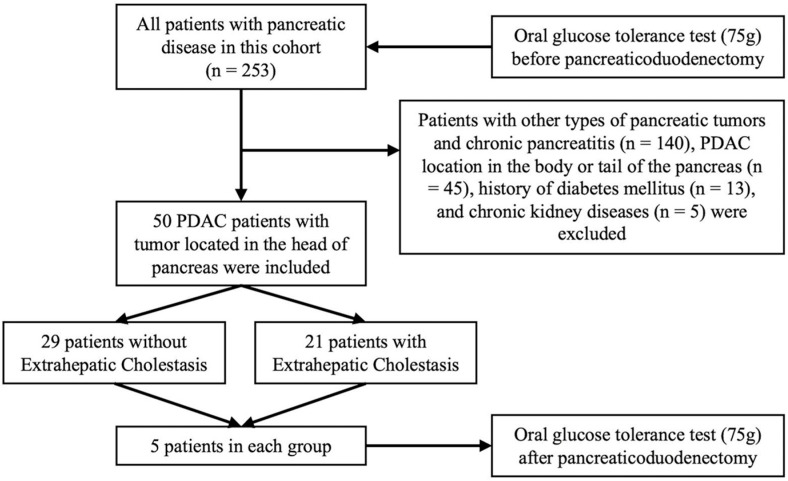
Flow chart of the study.

In this prospective cross-sectional study, from August 2017 to June 2018, 253 patients with pancreatic diseases were screened for abnormalities in glucose metabolism before pancreatic surgery. We excluded 140 patients diagnosed with other types of pancreatic tumor and chronic pancreatitis, 45 PDAC patients with tumors located in the body or tail of the pancreas, 13 patients with a history of diabetes mellitus, and 5 patients with chronic kidney diseases. The remaining 50 patients with ductal adenocarcinoma of the pancreatic head were included for further analysis (Figure 1). All patients had normal kidney function, as determined by medical history, estimated glomerular filtration rate, and urinalysis. Extrahepatic cholestasis was diagnosed by imaging, suggesting an enlarged common bile duct due to the pancreatic head mass, total bilirubin level greater than 34.2 μmol/L, and direct bilirubin mainly increased. Accordingly, patients were classified into the extrahepatic cholestasis and no extrahepatic cholestasis groups. The duration of the patients with cholestasis ranged from one to four weeks. The tumor stage of PDAC patients was classified according to the 8th Edition American Joint Committee on Cancer staging system [16]. The diameter of the tumors and the dilation of the main pancreatic duct (MPD) were also recorded based on the CT images and the Intraoperative findings. This study was approved by the Biomedical Research Ethics Committee in our hospital (2014 Trail No.37) and complied with the terms of the Helsinki Declaration. Informed consent was obtained from all individual participants and/or legal guardians enrolled in the study.

### 2.2. Biochemical Parameters

A 3 h oral glucose tolerance test (OGTT) was performed with a 75 g glucose load for all included patients before PD and 5 patients, each with preoperatively elevated or normal bile acid levels after PD when bile acid levels returned to normal. Blood samples were taken at 0, 30, 60, 120, and 180 min time points, and the plasma glucose, insulin, and C-peptide concentrations of each sample were assayed. The plasma glucose was tested by the hexokinase method on a Cobas e702 analyzer (Roche, Mannheim, Germany). The plasm insulin and C-peptide concentrations were analyzed by the electrochemiluminescence immunoassay on a Cobas e601 analyzer (Roche, Germany). Meanwhile, HbA1c was measured using high-performance liquid chromatography in an HLC-723GX analyzer (Tosoh Corporation, Tokyo, Japan). Patients were classified as having normal glucose tolerance (NGT, fasting plasma glucose < 5.6 mmol/L and 2 h plasma glucose < 7.8 mmol/L), prediabetes mellitus (Pre-DM, fasting plasma glucose 5.6–6.9 mmol/L and/or 2 h plasma glucose 7.8–11 mmol/L), or new-onset diabetes (NOD, fasting plasma glucose ≥ 7 mmol/L and/or 2 h plasma glucose ≥ 11.1 mmol/L), according to the American Diabetes Association criterion [17].

The Matsuda index was adopted to measure insulin sensitivity [18]. To evaluate β-cell function corrected for the degree of insulin sensitivity, the insulinogenic index (IGI) and insulin secretion/insulin resistance index (ISSI-2, or disposition index) were calculated as previously described [19]. Hepatic insulin clearance (HIC) was calculated as the ratio of fasting C-peptide and fasting plasm insulin, and 2 h or 3 h postprandial HIC was calculated from the ratio of C-peptide AUC_0–120 min_ (area under the curve) and plasma insulin AUC_0–120 min_ or AUC_0–180 min_ [20].

Conjugated and unconjugated bilirubin, total bile acids, alanine aminotransferase, aspartate transaminase, alkaline phosphatase, gamma-glutamyl transferase, albumin, triglyceride, total cholesterol, high-density lipoprotein, and low-density lipoprotein were measured in the laboratory medicine department according to the national standards. Total bile acid levels over 10 μmol/L, triglyceride levels over 1.88 mmol/L, and high-density lipoprotein levels less than 0.90 mmol/L were abnormal.

### 2.3. Statistical Analysis

Data were presented as frequencies for categorical variables and analyzed by Pearson’s Chi-square test or Fisher’s exact test. Continuous variables were expressed as mean ± standard deviation and were analyzed using the independent samples *t*-test or Mann–Whitney U nonparametric test. Relationships between variables were estimated by linear regression analysis and nonparametric Spearman correlation. The logistic regression models were used to compute the odds ratio (OR) with a 95% confidence interval (CI) estimate of relative risk. Factors with a *p*-value less than 0.100 in univariate analysis were involved in the multivariate analysis. A two-sided *p*-value less than 0.05 was regarded as statistically significant. All the data were analyzed by the SPSS version 24.0 (IBM, New York, NY, USA).

## 3. Results

### 3.1. Clinical and Metabolic Characteristics

The clinical and metabolic characteristics of included patients are summarized in Table 1. The prevalence of NGT, Pre-DM, and NOD in these patients was 26%, 36%, and 38%, respectively. Patients with extrahepatic cholestasis more frequently suffered from worsened impaired glucose homeostasis (Pre-DM and NOD) than patients without extrahepatic cholestasis (95.2% [20/21] and 58.6% [17/29], *p* = 0.004) before PD. Plasma total bile acid levels (93.8 ± 93.0 μmol/L vs. 5.1 ± 5.9 μmol/L), along with triglyceride (2.1 ± 1.2 mmol/L vs. 1.3 ± 0.5 mmol/L), were higher in extrahepatic cholestasis patients (both *p* values < 0.05). In contrast, high-density lipoprotein (0.5 ± 0.4 mmol/L vs. 1.2 ± 0.4 mmol/L) and low-density lipoprotein (1.9 ± 1.0 mmol/L vs. 2.4 ± 0.7 mmol/L) were lower (both *p* values < 0.05). Dilation of the main pancreatic duct occurred in 76% (38/50) of the included patients and showed no statistically significant difference between the two groups.

### 3.2. Risk Factors for Impaired Glucose Homeostasis before PD

To identify the risk factors for developing impaired glucose homeostasis in ductal adenocarcinoma of pancreatic head patients, variables including age, sex, body mass index, tumor diameter, dilation of the MPD, plasma total bile acid, triglyceride, high-density lipoprotein, alanine aminotransferase, and unconjugated bilirubin levels were included in the logistic regression models. Univariate and multivariate analysis revealed that (Table 2) only elevated bile acid level was recognized as an independent risk factor (*p* = 0.024, OR = 6.85, 95% CI: 1.29–36.25). No correlation was found between the dilation of MPD and the impaired glucose homeostasis in ductal adenocarcinoma of pancreatic head patients.

### 3.3. Glucose Metabolism Traits in Ductal Adenocarcinoma of Pancreatic Head Patients with Elevated Bile Acid

**Figure 2:**
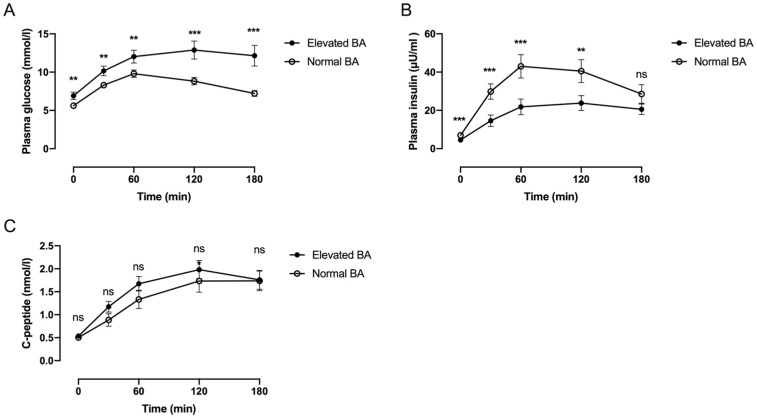
(**A**) Plasma glucose, (**B**) plasma insulin, and (**C**) C-peptide levels during the OGTT test in the Elevated-BA group (black circles) and the Normal-BA group (white circles). Data are means ± SE. ** *p* < 0.01; *** *p* < 0.001; ns, no significance.

To investigate the glucose metabolism traits in ductal adenocarcinoma of pancreatic head patients with elevated bile acid, we demonstrate the changes in OGTT-based curves and glucose metabolic index in patients with different bile acid levels. Compared with patients with normal bile acid levels, fasting and postprandial plasma glucose levels were higher in patients with elevated bile acid levels (all *p* values < 0.05, Figure 2 and Table 3). In contrast, fasting and postprandial plasma insulin levels were lower (all *p* values < 0.05). There was no difference in the plasma C-peptide levels (all *p* values > 0.05).

**Figure 3:**
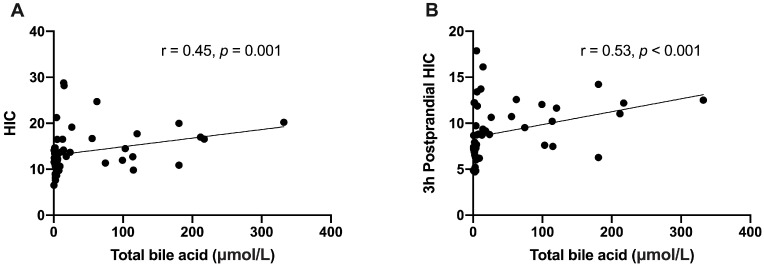
Correlation between bile acid levels and (**A**) HIC and (**B**) 3 h postprandial HIC. HIC, hepatic insulin clearance.

Although both IGI (8.7 ± 8.9 vs. 3.3 ± 3.2, *p* = 0.005) and ISSI-2 (6.9 ± 3.5 vs. 4.4 ± 2.9, *p* = 0.005) were higher in the normal bile acid group, considering there was no difference in the plasma C-peptide levels (all *p* values > 0.05, Figure 2 and Table 3), elevated bile acid level might not impair the beta-cell function. The Matsuda index showed no statistical difference between the two groups (163.8 ± 98.9 vs. 147.1 ± 127.6, *p* = 0.619). This revealed that insulin resistance was not changed among ductal adenocarcinoma of pancreatic head patients with elevated or normal bile acid levels. For the glucose metabolism indexes reflecting insulin clearance functions of the liver, HIC (16.7 ± 5.3 vs. 11.7 ± 3.0) and 3 h postprandial HIC (10.6 ± 2.4 vs. 7.9 ± 3.1) were significantly higher in patients with elevated bile acid levels (both *p* values = 0.001, Table 3). The analysis of all the patients revealed strong positive correlations between bile acid levels and HIC (r = 0.45, *p* = 0.001) and 3 h postprandial HIC (r = 0.53, *p* < 0.001, Figure 3). 

### 3.4. Alteration of Glycemic Traits after Bile Acid Metabolism Normalized

**Figure 4:**
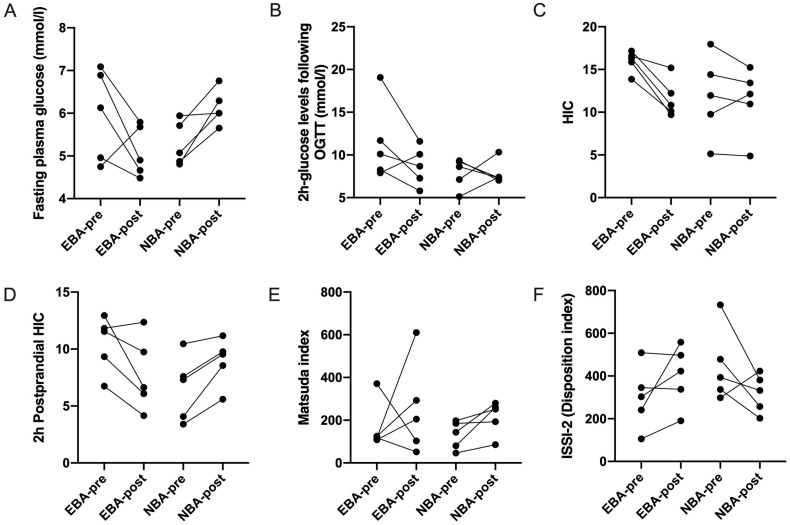
Changes in (**A**) fasting plasma glucose, (**B**) 2 h glucose levels following OGTT, (**C**) HIC, (**D**) 2 h postprandial HIC, (**E**) Matsuda index, and (**F**) ISSI-2 in elevated bile acid patients between pre (EBA-pre) and post (EBA-post) PD, and in normal bile acid patients between pre (NBA-pre) and post (NBA-post) PD. OGTT, oral glucose tolerance test; HIC, hepatic insulin clearance; PD, pancreaticoduodenectomy; ISSI-2, insulin secretion/insulin resistance index.

We performed another OGTT after PD for five patients with preoperative elevated bile acids when bile acid levels returned to normal (179.8 ± 100.8 vs. 6.0 ± 1.4 μmol/L) and for five patients with preoperative normal bile acid (Figure 4). Two preoperative elevated bile acid patients were relieved from impaired glucose tolerance. However, two normal bile acid patients with preoperative NGT developed impaired glucose tolerance after PD. A decreasing trend was observed when comparing preoperative and postoperative fasting and 2 h postprandial plasma glucose levels, HIC, and 2 h postprandial HIC levels in four of five patients with elevated bile acid levels, but not in preoperative normal bile acid patients (*p* < 0.05, Figure 4A–D). No clear tendency was found in the Matsuda index and ISSI-2 (Figure 4E,F).

## 4. Discussion

Our findings suggest that the prevalence of Pre-DM and NOD is high in PDAC patients. Ductal adenocarcinoma of the pancreatic head caused by extrahepatic cholestasis exacerbates this impaired glucose homeostasis through elevated bile acid levels. Mechanically, OGTT-based curves and glucose metabolic indexes reveal that the elevated HIC in patients with elevated bile acid could account for the decrease in plasma insulin and then result in the increase in plasma glucose levels. Preoperative elevated bile acid patients experienced a tendency to decrease in fasting and 2 h postprandial blood glucose levels accompanied by a decrease in HIC levels after PD, which further verified our assumption that elevation in HIC might be the reason for the transient worsened impaired glucose homeostasis in ductal adenocarcinoma of pancreatic head patients with elevated bile acid.

To our knowledge, our study suggests a link between elevated bile acid and worsened impaired glucose homeostasis for the first time. Indeed, the relationship between bile acid and glucose tolerance status has been extensively investigated in pregnancy since elevated serum bile acid levels are also characteristic of intrahepatic cholestasis in pregnant women. Similar findings have been reported in a recent prospective cohort study that women with higher serum total bile acid levels during early mid-pregnancy have a 1.78-fold increased risk of developing gestational diabetes mellitus [21]. The interrupted bile acid enterohepatic cycling by extrahepatic and intrahepatic cholestasis results in elevated plasma bile acid being predominantly primary bile acids, whereas secondary bile acids, which need to be formed by the gut microbiota, tend to reduce [22]. In this case, the increase in β-muricholic acid, one of the primary bile acids, was identified as the metabolic marker for the diagnosis of gestational diabetes [23]. Conversely, the decline in the secondary bile acids, glycoursodeoxycholic acid and deoxycholic acid, in early pregnancy was independently associated with an increased risk of gestational diabetes mellitus in Chinese pregnant women [24,25].

Insulin immediately enters the liver via the abdominal portal vein when secreted by the beta-cells of the pancreas. The liver is the primary site of insulin clearance, and over half of the portal insulin is removed during first-pass transit. Recent studies have emphasized the importance of modeling insulin clearance rather than secretion only [26]. In our study, patients with elevated bile acid had reduced insulin levels accompanied by a decrease in IGI and ISSI-2 based on the OGTT. However, there was no difference in C-peptide levels compared with patients with normal bile acid levels. This might reflect comparable insulin secretion functions in the two groups. Therefore, the decrease in serum insulin levels might be mainly caused by the increased HIC and the 3 h postprandial HIC, which reflects the insulin clearance function of the liver.

The insulin-degrading enzyme is thought to be a major enzyme responsible for insulin clearance in the liver [27]. However, previous works have shown that bile acids exert opposite effects on insulin-degrading enzymes in the context of different diseases. On the one hand, an animal study has verified that taurine-conjugated bile acid could increase insulin-degrading enzyme expression in the liver through the hepatic membrane bile acid receptor, sphingosine-1-phosphate receptor 2, and then improved insulin clearance in the high-fat diet mice model [28]. On the contrary, in the early stage of the type 1 diabetes mice model, taurine-conjugated bile acid could reduce insulin degradation [29]. Despite a strong positive correlation found between bile acid and HIC in our study, the exact effect of bile acids in humans needs further investigation.

Although we observed no difference in insulin secretion function in ductal adenocarcinoma of pancreatic head patients with different bile acid levels, bile acids could impair beta-cell function. A recent study provided insight into beta-cell dysfunction induced by extrahepatic cholestasis in periampullary tumor patients, which might mediate by the impaired beta-cell glucagon and glucagon-like peptide 1 sensitivity and the concomitant reduction in glucose-dependent insulinotropic polypeptide production in response to meals [10]. Indeed, bile acids could activate the Takeda G protein receptor 5 on intestinal L-cells and pancreatic alpha-cells to increase glucagon-like peptide 1 secretion, then promote glucose-stimulated insulin secretion of beta-cells [13,30]. Considering this contradiction, one limitation of our study is that the hyperglycemic clamp test was not performed to further validate the beta-cell function.

In an elegant study, the fasting plasma glucose level was found to increase with tumor volume [31]. However, tumor size was not identified as a risk factor for impaired glucose homeostasis in our study. That might be explained by not including sufficient patients with earlier tumor stages. The diameter of tumors in our cohort was all over one centimeter, whereas the smallest tumor volume associated with relative hyperglycemia was 1.1 to 2.0 cubic centimeters [31]. This is also reflected in the high prevalence of impaired glucose homeostasis in our cohort. 

What is more, using OGTT instead of fasting plasma glucose to diagnose pre-DM and NOD might also have some impact. Apparently, our study does not mean that bile acid, but not tumor size is the risk factor; rather, elevated bile acid is associated with worsened impaired glucose homeostasis in ductal adenocarcinoma of pancreatic head patients. The obstruction of MPD could be generating a malfunction of the entire pancreas islet; however, the tumor-induced dilation of MPD was not identified as a risk factor for impaired glucose homeostasis in the present study. When comparing the glucose metabolism indexes reflecting beta-cell function between patients with or without dilation of the MPD, both the IGI and the ISSI-2 showed no significant difference between groups. It is possible that in ductal adenocarcinoma of pancreatic head patients, the changes in bile acid metabolism caused by extrahepatic cholestasis affect glucose metabolism faster than the beta-cell decrease caused by main pancreatic duct obstruction. Considering the beta-cell functional reserve, beta-cell dysfunction will be induced only when the beta-cell decrease caused by MPD obstruction reaches a certain degree. Remnant pancreas volume might be an indicator of the degree of beta-cell decrease, compared with the obstruction of MPD. Recent studies provide insight into the correlation between pancreas volume and diabetes. Pancreas volume is recognized as a novel non-invasive biomarker to predict the progression of type 1 diabetes [32,33]. Similar results were also reported in a Mendelian randomization study demonstrating a causal effect of pancreatic volume on the reduced risk of type 2 diabetes [34]. The relationship between remnant pancreas volume and diabetes secondary to PDAC needs further investigation.

Similar to bariatric surgery, the altered gastrointestinal tract after PD could improve insulin resistance reflected by the homeostasis model assessment of insulin resistance [3,4]. The trend of a better insulin resistance level revealed by the Matsuda index was also found in the post-PD patients with normal bile acid levels. However, the fasting blood glucose level of these patients tends to increase, accompanied by the decreasing trend of ISSI-2, which might reflect the decrease of insulin secretion functions after PD. Another limitation of our study is that more patients should be involved to assess the alteration in glucose metabolism in ductal adenocarcinoma of pancreatic head patients with different bile acid levels after PD.

## 5. Conclusions

In conclusion, this prospective study suggested the connection between elevated bile acid levels and worsened impaired glucose homeostasis in ductal adenocarcinoma of pancreatic head patients. Increased HIC caused by elevated bile acid levels might be the mechanism of decreased plasma insulin levels. The relief of biliary obstruction after PD might explain the resolution of glucose tolerance. However, further validation is needed. If these findings are validated in all patients with extrahepatic cholestasis requiring Whipple surgery, these might provide clinical evidence for preoperative biliary drainage and bile reinfusion.

## Figures and Tables

**Table 1:** Clinical and metabolic characteristics of pancreatic head ductal adenocarcinoma patients with and without extrahepatic cholestasis.

Characteristics	Extrahepatic Cholestasis n = 21	No-Extrahepatic Cholestasis n = 29	*p* Value
Sex (Male)	18 (85.7)	18 (62.1)	0.110
Age (years)	62.9 ± 10.5	59.8 ± 11.6	0.340
BMI (kg/m^2^)	21.9 ± 2.1	22.3 ± 2.8	0.625
Dilation of MPD	16 (76.2)	22 (75.9)	>0.999
Tumor diameter	3.3 ± 1.1	2.9 ± 1.0	0.206
Tumor stage			0.551
I–II	12 (57.1)	20 (69.0)	
III–IV	9 (42.9)	9 (31.0)	
Total bilirubin (μmol/L)	174.4 ± 140.3	12.2 ± 6.1	<0.001
Conjugated bilirubin (μmol/L)	150.5 ± 120.6	5.2 ± 3.7	<0.001
Unconjugated bilirubin (μmol/L)	23.9 ± 22.0	7.0 ± 3.2	0.002
Albumin (g/L)	37.9 ± 5.9	43.8 ± 4.3	<0.001
ALT (IU/L)	144.1 ± 158.3	24.3 ± 28.8	<0.001
ALP (IU/L)	378.0 ± 190.8	90.9 ± 59.6	<0.001
GGT (IU/L)	458.1 ± 500.8	41.23 ± 78.0	0.001
Total bile acid (μmol/L)	93.8 ± 93.0	5.1 ± 5.9	<0.001
Triglyceride (mmol/L)	2.1 ± 1.2	1.3 ± 0.5	0.009
Total cholesterol (mmol/L)	4.4 ± 1.5	4.1 ± 0.8	0.401
HDL (mmol/L)	0.5 ± 0.4	1.2 ± 0.4	<0.001
LDL (mmol/L)	1.9 ± 1.0	2.4 ± 0.7	0.033
Urea (mmol/L)	4.2 ± 1.5	4.3 ± 1.2	0.795
Creatine (μmol/L)	72.8 ± 17.4	68.1 ± 17.2	0.347
Fasting glucose levels (mmol/L)	7.0 ± 2.1	5.6 ± 0.9	0.007
2 h postprandial plasma glucose (mmol/L)	12.7 ± 5.4	8.9 ± 2.8	0.008
HbA1c (%)	6.4 ± 1.4	5.9 ± 0.8	0.173
Glucose tolerance status			0.004 *
Normal glucose tolerance	1 (4.8)	12 (31.0)	
Prediabetes mellitus	9 (42.8)	9 (44.8)	
New-onset diabetes	11 (52.4)	8 (27.6)	

Abbreviations: ALT, alanine aminotransferase; ALP, alkaline phosphatase; AST, aspartate aminotransferase; BMI, body mass index; GGT, gamma-glutamyl transferase; HDL, high-density lipoprotein; LDL, low-density lipoprotein; MPD, main pancreatic duct. * Fisher’s exact test between normal glucose tolerance and impaired glucose homeostasis (prediabetes mellitus and new-onset DM).

**Table 2:** Univariate and multivariate analysis for the risk factor of ductal adenocarcinoma of the pancreatic head patients developing impaired glucose homeostasis.

Variables	Univariate Analysis		Multivariate Analysis	
	*p* Value	OR (95% CI)	*p* Value	OR (95% CI)
Sex				
Female	Reference		-	-
Male	0.796	1.20 (0.30–4.79)	-	-
Age (years)				
<69	Reference		-	-
≥69	0.426	1.81 (0.42–7.74)	-	-
BMI (kg/m^2^)				
<23.9	Reference		Reference	
≥23.9	0.098	0.32 (0.08–1.23)	0.159	0.08 (0.08–1.50)
Dilation of MPD				
No	Reference			
Yes	0.928	0.93 (0.21–4.15)		
Tumor diameter (cm)	0.219	1.53 (0.78–2.99)		
Tumor stage				
I–II	Reference		-	-
III–IV	0.266	2.27 (0.53–9.67)	-	-
Unconjugated bilirubin (μmol/L)				
<17.0	Reference			
≥17.0	>0.999	-		
Bile acid (μmol/L)				
<10.0	Reference		Reference	
≥10.0	0.018	7.22 (1.34–37.25)	0.024	6.85 (1.29–36.25)
Triglyceride (mmol/L)				
<1.88	Reference		-	-
≥1.88	0.944	0.95 (0.24–3.76)	-	-
HDL (mmol/L)				
≥0.9	Reference		-	-
<0.9	0.269	2.13 (0.56–8.16)	-	-
ALT (IU/L)				
<40	Reference		-	-
≥40	0.118	3.16 (0.75–13.36)	-	-

Abbreviations: ALT, alanine aminotransferase; BMI, body mass index; HDL, high-density lipoprotein; MPD, main pancreatic duct; OR, odds ratio; CI, confidence interval.

**Table 3:** Univariate and multivariate analysis for the risk factor of ductal adenocarcinoma of the pancreatic head patients developing impaired glucose homeostasis.

Characteristics	Elevated Bile Acids n = 21	Normal Bile Acids n = 29	*p* Value
Glucose AUC_0–180 min_	40.4 ± 14.3	29.9 ± 7.0	0.005
Insulin AUC_0–180 min_	68.1 ± 47.4	121.9 ± 78.3	0.007
C-peptide AUC_0–180 min_	4.72 ± 2.92	5.55 ± 2.57	0.298
Matsuda index	163.8 ± 98.9	147.1 ± 127.6	0.619
Insulinogenic index	3.3 ± 3.2	8.7 ± 8.9	0.005
ISSI-2	4.4 ± 2.9	6.9 ± 3.5	0.010
HIC	16.7 ± 5.3	11.7 ± 3.0	0.001
3 h postprandial HIC	10.6 ± 2.4	7.9 ± 3.1	0.001

Abbreviations: AUC, area under the curve; ISSI-2, insulin secretion/insulin resistance index; HIC, hepatic insulin clearance.

## Data Availability

Data supporting the findings in this study are available within this article and from the corresponding author upon reasonable request.
